# Biostimulant-Based Molecular Priming Improves Crop Quality and Enhances Yield of Raspberry and Strawberry Fruits

**DOI:** 10.3390/metabo14110594

**Published:** 2024-11-05

**Authors:** Petar Kazakov, Saleh Alseekh, Valentina Ivanova, Tsanko Gechev

**Affiliations:** 1 Center of Plant Systems Biology and Biotechnology, 14 Knyaz Boris I Pokrastitel Str., 4023 Plovdiv, Bulgaria; kazakov@cpsbb.eu (P.K.); alseekh@cpsbb.eu (S.A.); vivanova@cpsbb.eu (V.I.); 2 Max Planck Institute of Molecular Plant Physiology, 1 Am Muehlenberg, 14476 Potsdam, Germany; 3 Department of Molecular Biology, Plovdiv University, 24 Tsar Assen Str., 4023 Plovdiv, Bulgaria

**Keywords:** *Ascophyllum nodosum*, biostimulants, LC-MS, ICP-MS, raspberry, strawberry

## Abstract

Background/Objectives: The biostimulant SuperFifty, produced from the brown algae *Ascophyllum nodosum*, can improve crop quality and yield and mitigate stress tolerance in model and crop plants such as *Arabidopsis thaliana*, pepper, and tomato. However, the effect of SuperFifty on raspberries and strawberries has not been well studied, especially in terms of nutritional properties and yield. The aim of this study was to investigate the effect of SuperFifty on the quality and quantity of raspberry and strawberry fruits, with a focus on metabolic composition and essential elements, which together determine the nutritional properties and total yield of these two crops. Methods: Metabolome analysis was performed by liquid chromatography–mass spectrometry analysis (LC-MS), and essential elements analysis was performed by inductively coupled plasma-mass spectrometry (ICP-MS). Results: Here, we demonstrate that SuperFifty increases the fruit size of both raspberries and strawberries and enhances the yield in these two berry crops by 42.1% (raspberry) and 33.9% (strawberry) while preserving the nutritional properties of the fruits. Metabolome analysis of 100 metabolites revealed that antioxidants, essential amino acids, organic acids, sugars, and vitamins, such as glutathione, alanine, asparagine, histidine, threonine, serine, tryptophan, sucrose, citric acid, pantothenic acid (vitamin B5), as well as other primary metabolites, remain the same in the SuperFifty-primed fruits. Secondary metabolites, such as caffeic acid, p-coumaric acid, kaempferol, and quercetin, also maintained their levels in the SuperFifty-primed fruits. Analysis of essential elements demonstrated that elements important for human health, such as Zn, Mn, Fe, B, Cu, K, and Ca, maintain the same levels in the raspberry and strawberry fruits obtained from the biostimulant-primed plants. Magnesium, an important element known as a co-factor in many enzymatic reactions related to both plant physiology and human health, increased in both raspberry and strawberry fruits primed with SuperFifty. Finally, we discuss the potential financial and health benefits of the SuperFifty-induced priming for both growers and consumers. Conclusions: We demonstrate that SuperFifty significantly enhances the yield of both raspberries and strawberries, improves the marketable grade of the fruits (larger and heavier fruits), and enhances the nutritional properties by elevating Mg content in the fruits. Altogether, this biostimulant-induced molecular priming offers an environmentally friendly, efficient, and sustainable way to enhance the yield and quality of berry crops, with clear benefits to both berry producers and customers.

## 1. Introduction

Plant biostimulants are organic molecules, plant extracts, or microorganisms that can stimulate plant growth and development [1,2]. Furthermore, some of them can protect from unfavorable environmental conditions and enhance yields without compromising on quality [3,4]. Treatment with biostimulants can also mitigate subsequent abiotic or oxidative stress by inducing endogenous molecular plant defense mechanisms, a process known as molecular priming [3].

Small organic molecules such as acetate, GABA, hydrogen peroxide, etc., can induce genes related to growth, development, and stress responses, resulting in improved growth/development or resilience to stresses [3]. As naturally occurring substances, plant-derived biostimulants are non-toxic, biodegradable, and considered eco-friendly tools to enhance crop productivity [1].

The biostimulant SuperFifty (SF) is obtained from the brown algae *Ascophyllum nodosum* (L.) Le Jolis and is harvested in a sustainable way from the Atlantic Ocean [1]. SF is rich in fucoidan, laminarin, and alginates and is produced by and commercially available from the Irish company BioAtlantis.

Previously, it was demonstrated that SF completely abolishes oxidative stress in the model plant *Arabidopsis thaliana* and the crop plants pepper and tomato, fully preventing the oxidative stress-induced leaf lesions and preserving the intactness and functionality of the photosystems [5]. In *A. thaliana*, the protection from oxidative stress was concomitant with global transcriptional reprogramming and metabolome reconfiguration, which together restored the molecular signature of the non-stressed plants [6]. In addition, extracts from *A. nodosum* were shown to mitigate drought stress in okra and soybean, as well as freezing tolerance in *A. thaliana* [7,8,9]. Hence, the main benefits of biostimulants are (i) they are ecologically friendly and biodegradable, (ii) they are obtained from renewable sources that can boost a crop performance by stimulating growth, and (iii) they play a vital role in mitigating stress and improving nutrient assimilation [1].

Berry crops, such as raspberry (*Rubus idaeus* L.) and strawberry (*Fragaria × ananassa* (Duchesne ex Weston) Duchesne ex Rozier), are important ingredients in the human diet and are considered healthy food because of their rich nutritional properties. They contain a wealth of antioxidants, flavonoids, phenolics, carotenoids, and ascorbic acid, as well as other vitamins and elements important for human health [10,11,12]. Raspberries are richer in vitamins and elements, providing three times more vitamins K, E, B5, and Zn, while strawberries are twice as rich in vitamin C. Due to these nutrition- and health-related benefits and an increase in awareness, the consumption of the two berries in the USA has recently increased by 192% for raspberries and by 45% for strawberries [13]. Overall, both the production and the consumption of these berry fruits have increased globally. While there are a number of studies investigating the effects of the *Ascophyllum nodosum*-derived biostimulants on vegetable crops, studies of seaweed biostimulants on berry crops are rather limited. Commercial extracts from *Durvillaea potatorum* and *A. nodosum* were shown to stimulate the growth of strawberry roots and positively influence yield [14]. However, no comprehensive metabolome studies were conducted on strawberries in order to evaluate the metabolites and the essential minerals, which are of importance to both plant physiology and human health. The research on raspberries is even more limited, with one study showing an increase in Brix by seaweed extracts. However, how seaweed biostimulants affect the metabolic composition and nutritional properties of raspberry fruits has been completely unknown.

Here, we evaluated the effect of molecular priming by SF on elite high-yielding cultivars of raspberry and strawberry crops. The aim was to evaluate the nutritional properties of raspberry and strawberry fruits originating from SF-treated plants (metabolic composition, including sugars, amino acids, organic acids, some vitamins, and secondary metabolites of plant and human health significance; essential minerals analysis), as well as marketable grade of the fruits and total yield. In the case of yield enhancement, we also determined the potential financial benefits for the producers, considering the current market values of raspberries and strawberries.

## 2. Materials and Methods

### 2.1. Plant Material, Growth, Biostimulant Treatment, and Measures of Fruit Weight and Yield

Raspberry (Rubus idaeus cv. Vajolet) and strawberry (*Fragaria* × *ananassa* cv. Elsanta) plants were obtained from the Bulgarian Raspberry and Berries Association and grown on soil in climate-controlled greenhouses with a temperature of 25 °C and relative humidity of 70%. The flowers of both crops were pollinated with Bombus spp., which is available commercially and purchased from Opora Zaden Ltd., Tsalapitsa, Bulgaria. One hundred strawberry plants and forty raspberry plants were used for the experiment. Five biological replicates from different plants were taken for molecular analysis. SF is produced by BioAtlantis Ltd., Tralee, Kerry, Ireland., and is commercially available. Plants were sprayed with a 0.4% solution of SF or with water at flower bud emergence and flowering six times over the period of three months, and fresh fruits were harvested weekly in the period of three months. This concentration was chosen based on our previous experiments using this biostimulant on other plants (A. thaliana, eggplant, pepper, tomato), which demonstrated the best effect of 0.4% (unpublished results). Lower concentrations were less effective, and higher concentrations were too expensive for the farmers. The weight of 50 individual fresh raspberry and strawberry fruits was measured with an analytical balance, and the average weight and standard deviation were calculated. Statistical analysis was performed using the R package for statistical computing, with which the standard deviation, standard error of the mean, and the *p*-values between control and SF-treated samples were calculated. The yield was calculated by summing up the weight of all weekly harvests in the period of three months.

### 2.2. Metabolome Analysis of Primary Metabolites by LC-MS

Raspberry and strawberry fruits were harvested and immediately frozen in liquid nitrogen to preserve the state of metabolites. Five biological replicates were taken for both raspberries and strawberries. Samples from freshly collected fruits were immediately frozen in liquid nitrogen and then ground to a fine powder, and then secondary metabolite analysis by LC–MS was performed as described previously [15]. The relative metabolite abundances were defined as the normalized peak area for each metabolite; peaks were normalized to the internal standard (Isovitexin) and sample weight. All data were processed using Xcalibur 2.1 software (Thermo Fisher Scientific, Waltham, MA, USA). The obtained data matrix of peak area was normalized using the internal standard isovitexin, CAS: 29702-25-8. Metabolite identification and annotation were performed using metabolite databases [16]. Relative metabolite abundances of detected compounds are shown in Supplemental Table S1.

### 2.3. Analysis of Elements by ICP-MS

Fruits from control and SuperFifty-primed raspberry and strawberry plants were immediately frozen in liquid nitrogen, then ground to a fine powder, and prepared for ICP-MS analysis. Measurements were performed using an inductively coupled plasma-mass spectrometer (7850 ICP-MS, Agilent Technologies, Santa Clara, CA, USA). The system was fitted with a glass nebulizer, quartz spray chamber, nickel cones, and a torch with a 2.5 mm injector, as well as an Ultra High Matrix Introduction system and ORS4 cell operating in helium (He) mode. The optimized ICP-MS operation conditions for analysis were as follows: RF power of 1600 W, plasma argon flow rate of 15.0 L min^−1^, and nebulizer gas flow rate of 0.9 L min^−1^. Data acquisition was performed using spectrum analysis and full quantitative mode. The tuning was performed with a 10 μg L^−1^ solution (Ce, Co, Li, Tl, and Y). An ICP Multielement Standard Solution IV Certipur^®^ (Merck, Rahway, NJ, USA) was used to prepare calibration curves and QC standards for the quantitative analysis of the elements. The determination coefficient of the calibration curves for all elements was higher than 0.9991. A MARS 6 microwave digestion system (CEM Corporation, Matthews, NC, USA) was used for sample preparation. The digestion was performed in PTFE vessels by adding 0.5 mL trace metal grade concentrated HNO_3_ and 2 mL 30% H_2_O_2_ to 0.25 g homogenized and lyophilized samples. The samples were left for 30 min before being placed in the microwave and digested in closed vessels as described [17]. Samples and method blanks were prepared and digested in a single batch and later diluted to 15 mL with reagent water.

## 3. Results

### 3.1. The Ascophyllum Nodosum-Derived Biostimulant SuperFifty Increases Fruit Size, Weight, and Yield in Raspberry and Strawberry Plants

**Figure 1:**
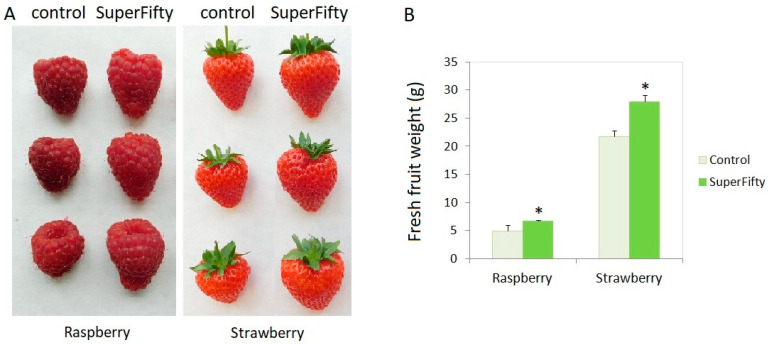
SuperFifty enhances the size and weight of berry fruits. (**A**) Representative pictures of raspberry (**left**) and strawberry (**right**) fruits from plants treated with water (control) or SuperFifty. (**B**) The data are the average weight of 50 fruits treated with water and SuperFifty, mean values +/− SEM. Asterisks indicate statistically significant differences from controls (*p* < 0.01).

Raspberry and strawberry plants were grown in climate-controlled greenhouses and sprayed with SF, as described in Materials and Methods. Fruits were harvested weekly for a period of three months and examined for weight, size, and overall yield. The number of fruits per plant was the same in control and SF-treated plants. The relative water content of the fruits from the control and SF-treated plants was also the same. However, the size, weight, and total yield of the SF-treated plants were higher than the control plants. The weight of individual fruits was measured by an analytical balance after each harvest, and the total yield was calculated by summing up the weights of all harvests. The size and weight of the raspberry and strawberry fruits are presented in Figure 1. The greenhouse experiments with SF-treated raspberries and strawberries showed that the fruits of the berry crops became larger and heavier (Figure 1), and the total yield increased by 42.1% in raspberries and by 33.9% in strawberries (Table 1).

Plants were either mock-treated (water) or treated with a 0.4% *v*/*v* aqueous solution of SuperFifty at the flowering and fruit set stages, and the harvest was collected multiple times. The numbers given in the table are grams per 20 raspberry plants and per 50 strawberry plants. The data were collected from six harvests in a period of three months.

### 3.2. Nutritional Properties of Berry Fruits, Evaluated by GC-MS and ICP-MS Analyses of Primary Metabolites and Essential Elements, Are Preserved in the Biostimulant-Primed Plants

**Figure 2:**
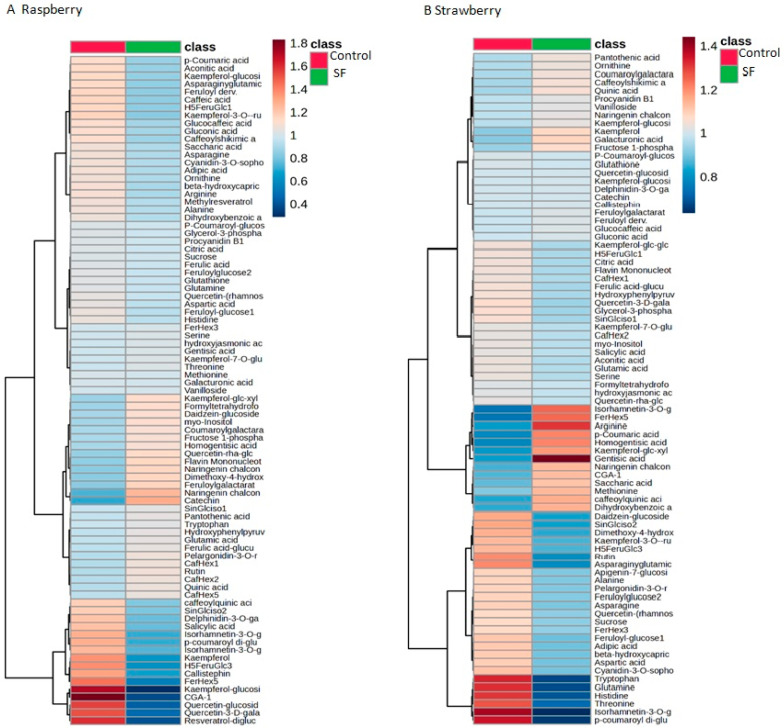
LC-MS analysis of primary metabolites in raspberry and strawberry fruits after SuperFifty-induced molecular priming. Red color and blue color indicate higher and lower relative metabolic abundances, respectively. The heatmap is based on data from five biological replicates.

In order to evaluate the nutritional properties of the raspberries and strawberries, as well as to determine if SF priming affected those traits, we performed an evaluation of primary and secondary metabolites as well as elements essential for both plant physiology and human health (Figure 2, Supplementary Table S1). The group of primary metabolites included amino acids (e.g., alanine, arginine, aspartic acid, glutamine, histidine, methionine, ornithine, serine, threonine, tryptophan), essential sugars, and sugar derivatives (e.g., sucrose), organic acids (citric acid, galacturonic acid, gluconic acid), antioxidants, and vitamins (glutathione, pantothenic acid, etc.). Secondary metabolites included important intermediates of biochemical pathways, as well as metabolites with health-promoting properties (e.g., caffeic acid, p-coumaric acid, kaempferol, and quercetin). Most of the metabolites had the same abundances in control and in SF-treated plants (Figure 2, Supplementary Table S1). The minor differences observed in a few metabolites were not statistically significant.

The complex taste and aroma in both raspberry and strawberry fruits are determined by hundreds of primary and secondary metabolites. Table 2 presents the relative abundances of major metabolites (sugars, organic acids, amino acids, antioxidants, vitamins) that contribute to the taste and nutritional properties of raspberry and strawberry fruits.

**Figure 3:**
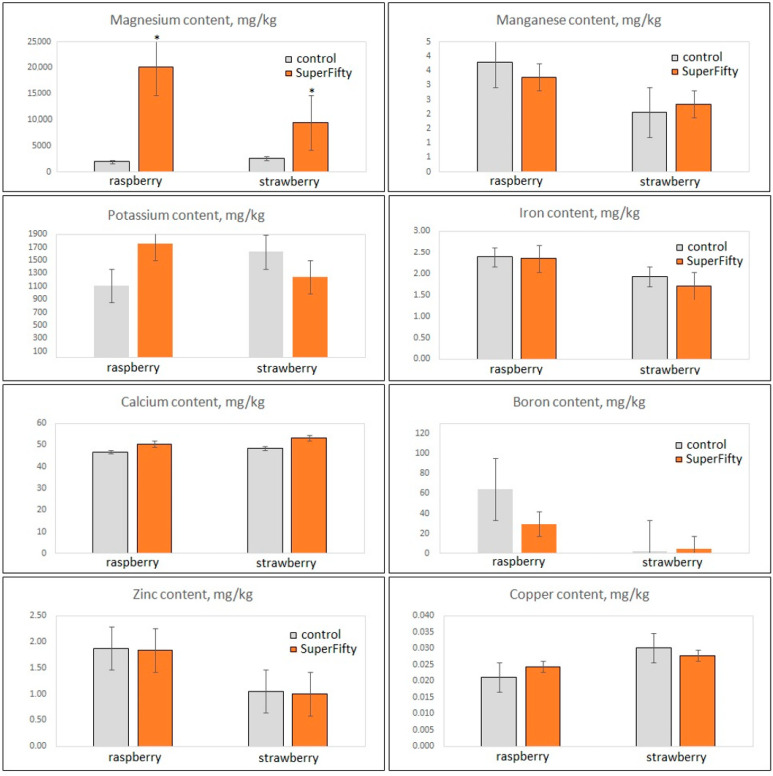
ICP-MS analysis of essential elements in raspberry and strawberry fruits after SuperFifty-induced molecular priming. The data are from five biological replicates ± SD. Asterisks indicate statistically significant differences from controls (*p* < 0.01).

In addition to the metabolome analyses, we performed ICP-MS analysis of elements that are essential micro- or macronutrients for plant physiology as well as for human health, including boron, calcium, copper, iron, magnesium, manganese, potassium, and zinc (Figure 3). Raspberries contained much more boron than strawberries; they also contained more zinc. All other elements had comparable abundances in the fruits of the two berry crops. The molecular priming by SF caused a substantial accumulation of magnesium in both raspberries and strawberries (Figure 3). The other essential elements were unaltered.

### 3.3. Market Benefit and Environmental Sustainability Analyses of the Molecular Priming Technology Applied to Raspberry and Strawberry Crops

**Figure 4:**
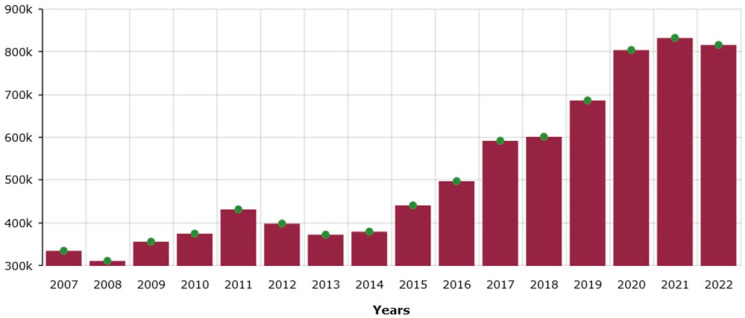
World raspberry production in 2007–2022 in thousands of metric tons. Source: International Raspberry Association (www.internationalraspberry.net/, accessed on 4 November 2024).

According to the International Raspberry Organization, comprising 13 countries which cumulatively account for more than 80% of the raspberry production in the World, the raspberry production exceeded 800,000 tons in 2022 and is growing every year (Figure 4; www.internationalraspberry.net/, accessed on 4 November 2024). The constant increase in raspberry production is driven by the high demand, making this the fastest-growing consumer fruit segment in many countries. Raspberries are considered a healthy food due to their rich mineral content, high amounts of antioxidants, flavonoids, vitamins, and other nutrients like sugars and organic acids [18]. The price of raspberry varies from EUR 141.94 per 100 kg (Bulgaria) to EUR 1142.47 per 100 kg (Lithuania) www.nationmaster.com/nmx/ranking/raspberries-producer-price, accessed on 4 November 2024). Assuming a constant application of the seaweed-induced molecular priming would increase the yield by 40% and an average price of EUR 400 per 100 kg (based on the production output in different countries (www.nationmaster.com/nmx/ranking/raspberries-producer-price, accessed on 4 November 2024), the increased revenue is calculated at EUR 1,280,000,000 for the global market: a colossal increase. Likewise, the global strawberry market is EUR 17,000,000,000, and a 30% biostimulant-driven increase in production can result in an additional EUR 5,100,000,000 in revenue. These figures are indicative of the potential financial benefits of SF-priming to farmers. Accurate calculations are difficult, as more field experiments over several years are needed to see how yield will increase under field conditions in a changing environment. Nevertheless, they show the colossal potential of SF as a priming agent and a way to produce healthy, affordable food. A likely scenario from the biostimulant-driven increased production of raspberries and strawberries could be a decrease in prices; a moderate decrease in consumer prices will still be profitable for the producers, especially if the use of conventional agrochemicals is minimized, and will definitely benefit the consumers/end users. These calculations do not include the additional health benefits coming from increased consumption of these berry fruits, which are considered healthy foods and can promote healthy living.

## 4. Discussion

Plant seaweed-derived biostimulants are changing modern agriculture, as they have the potential to secure healthy and sustainable food production while preserving the environment. Abiotic stresses such as drought and extreme salinity can cause up to 80% of the global yield losses. Seaweed biostimulants, such as those produced by the brown algae A. nodosum, can activate the natural genetic defense mechanisms of plants, a process referred to as molecular priming, and protect from adverse environmental conditions [3,6]. Metabolic reconfigurations by seaweed extracts have already been observed in maize under drought stress [19]. Moreover, they can stimulate growth, enhance the yield, and improve the marketable grade of the produce without compromising crop quality, therefore contributing to sustainable agriculture. For example, an extract from the brown algae Ecklonia maxima improved plant growth, soluble solids content, and nitrogen, potassium, and magnesium concentrations in strawberry fruits [20]. In raspberries, microbial biostimulants improved the antioxidant capacity of fruits [21]. In addition to the positive effect on growth, abiotic and oxidative stresses, a biostimulant from A. nodosum protected from biotic stress as well by suppressing the growth of powdery mildew in strawberries [22].

Here, we demonstrate that SuperFifty-induced molecular priming technology is highly efficient, as small amounts of SuperFifty lead to significant yield enhancement (42.1% in raspberries and 33.9% in strawberries). Furthermore, it results in improved marketable grade (larger fruits) and nutritional properties (higher Mg content). This benefits both the farmers/producers and the clients/end users, who will benefit from potentially lower consumer prices and increased health-stimulating properties of the fruits from primed crops. This is also an ecologically friendly technology, as the seaweed biostimulants are derived from 100% natural products: the brown algae A. nodosum, produced in a sustainable way by controlled harvesting from the Atlantic Ocean. This technology has the potential to reduce the use of traditional fertilizers and other agrochemicals. SF is non-toxic, 100% biodegradable, and widely and easily applicable to berries and other crops.

In our study, the A. nodosum-derived biostimulant SF enlarges raspberry and strawberry fruits, improving their marketable grade and enhancing the overall yield. At the same time, the nutritional properties of the raspberry and strawberry fruits are preserved, as evaluated by the abundance of essential amino acids, sugars, organic acids, antioxidants, and vitamins. This is the first comprehensive metabolome study on the effect of seaweed biostimulants on raspberry and strawberry fruit quality. The mineral content of essential micro- and macroelements such as Mn, Fe, B, K, Ca, B, and Zn is also preserved in the raspberry fruits harvested from the SF-treated plants. Moreover, the fruits from the SF-treated plants accumulate higher Mg levels. The reasons for the enlarged fruit size and the accumulation of Mg by SF in the two berry fruits are unknown. However, a Mg transporter gene was upregulated by SF in a previous study in A. thaliana [6]. Future comprehensive transcriptomic analysis by RNA-seq can show if growth-related genes or/and Mg-related genes (e.g., Mg transporters) are induced by SF, which could mechanistically explain the larger size and higher Mg content in SF-treated plants. Magnesium is one of the seven essential macroelements, an important co-factor in hundreds of enzymes (600 enzymatic reactions) and molecular processes, such as DNA and RNA synthesis, redox homeostasis, metabolism, protein synthesis, circadian clock, etc. [23,24]. It is of paramount importance for the function of neurons, muscles, blood pressure, and the immune system [23,24]. The United States Food and Nutrition Board recommends a daily Mg intake of 420 mg for men and 320 mg for women, which is provided by Mg-containing food or/and Mg pills, which are more and more popular nowadays [23]. However, many people (e.g., 60% of Americans) do not consume the recommended daily amount of Mg [25]—hence, consuming SF-primed berry fruits could be a pleasant solution to this problem.

While the positive effects of SF on yield, stress tolerance, and nutritional properties are clear, still little is known about the molecular genetic mechanisms controlled by SF. Our previous results showed that SF counteracts oxidative stress in A. thaliana [6]. As many abiotic stresses lead to oxidative stress (e.g., drought, salinity, extreme temperatures), protecting from oxidative stress will protect against abiotic stresses as well. Previous transcriptomic studies in A. thaliana confirmed that notion, showing that stress marker genes are upregulated by drought and oxidative stress but not in SF-treated plants [6]. Furthermore, SF-treated plants have higher expression of growth and photosynthesis-related genes, which can explain the better growth. Functional validation of some of these genes could reveal more about the mechanism of SF action. For example, knockout of the transcription factor ERF54 leads to the abolishment of the SF protection (Sujeeth N, unpublished results), indicating that ERF54 is part of SF signaling. Raspberry and strawberry homologs of these transcription factors may be responsible for SF-induced transcriptional reprogramming and metabolome reconfiguration. Future research could utilize such an approach to identify more genes involved in the SF-modulated signaling cascade. Alternatively, genome-wide association studies (GWAS) with biostimulants like SF can also identify genes that are likely players in the biostimulant-modulated signaling network. Large GWAS panels are already available for many major crops.

## 5. Conclusions

In conclusion, this study demonstrated the efficacy of the concept of seaweed-induced molecular priming for yield enhancement, marketable grade improvement, and biofortification of berry crops. The economic effect of this yield enhancement can be colossal, bearing in mind the prominent markets for raspberries and strawberries. Furthermore, the benefits can be not only for the growers/berry producers but for the customers as well; biostimulant-enhanced berry production might result in better prices for the customers, and consuming Mg-enriched berry fruits, as in the case of SF-primed raspberries and strawberries, is a pleasant way of providing the required intake of Mg. Thus, the priming by SF not only improves plant physiology and enhances yield but can also have benefits for human health.

## Figures and Tables

**Table 1:** Enhancement of yield by the biostimulant SuperFifty in raspberry and strawberry fruits.

Crop	Fruit Fresh Weight (g)		% Increase Due to SuperFifty
	Control (Unprimed)	SuperFifty-Primed	
Raspberry	2742.4	3897.2	42.1
Strawberry	16,943.2	22,686.92	33.9

**Table 2:** Relative abundances of major metabolites (sugars, organic acids, amino acids, antioxidants, vitamins) that contribute to the taste and nutritional properties of raspberry and strawberry fruits. The data are an average from five biological replicates. The complete datasets for all metabolites are given in Supplemental Table S1.

	Raspberry		Strawberry	
Metabolite	Control	SF	Control	SF
Sucrose	57,760.00	56,180.00	29,040.00	24,798.00
Serine	278.60	285.40	45.28	42.11
Alanine	596.60	510.80	344.40	287.40
Threonine	176.20	184.20	98.25	55.97
Asparagine	8672.00	7304.00	5256.00	4328.00
Ornithine	134.80	112.87	5.50	5.96
Aspartic acid	6048.00	5466.00	3784.00	3076.00
Glutamine	2036.00	1906.80	2419.40	1239.40
Glutamic acid	10,314.00	11,324.00	4044.00	3744.00
Histidine	253.60	224.80	140.88	74.51
Glycerol-3-phosphate	195.60	188.00	110.47	97.88
Aconitic acid	84.16	63.71	138.79	128.23
Arginine	530.20	436.60	2.95	4.72
myo-Inositol	10,970.00	13,824.00	19,740.00	18,520.00
Citric acid	86,780.00	83,140.00	53,680.00	49,080.00
Quinic acid	369.20	424.60	1029.40	1149.20
Galacturonic acid	2124.00	2114.00	2172.00	2596.00
Gluconic acid	10,422.00	8274.00	6806.00	6898.00
Saccharic acid	5442.00	4588.00	1430.00	1818.00
Fructose 1-phosphate	2888.00	3500.00	3484.00	4008.00
Asparaginyglutamic acid	174.40	129.14	379.20	249.40
Glutathione	328.20	307.80	615.80	605.20
Caffeic acid	33.44	24.44	0.00	0.00
Methionine	7.62	7.53	0.57	0.71
Tryptophan	274.60	291.80	824.80	415.00
Pantothenic acid	473.80	514.40	39.95	43.51
Catechin	225.60	416.40	5850.00	5864.00
Salicylic acid	173.20	109.25	304.40	287.60
Vanilloside	381.20	383.80	4.18	4.39
Glucocaffeic acid	411.60	327.00	30.08	30.96
Resveratrol-diglucoside	21.55	5.39	0.00	0.00
Procyanidin B1	2366.00	2282.00	418.20	439.00
Formyltetrahydrofolate	9.76	12.42	37.58	36.39
Rutin	35.55	41.62	6.69	4.43
Ferulic acid	13.21	12.56	0.00	0.00
Kaempferol	2.42	1.14	11.16	13.22

## Data Availability

The original contributions presented in the study are included in the article/Supplementary Materials; further inquiries can be directed to the corresponding author/s.

## Supplementary Materials

The following supporting information can be downloaded at: https://www.mdpi.com/article/10.3390/metabo14110594/s1, Table S1: Relative metabolite abundances in raspberry and strawberry fruits.
